# Supplementary material for: Maternal Sepsis in Italy: A Prospective, Population-Based Cohort and Nested Case-Control Study
Source: Microorganisms. 2022 Dec 31;11(1):105. doi: 10.3390/microorganisms11010105 (PMC9865500; doi:10.3390/microorganisms11010105)
Supplement: Supplementary file 1 [file microorganisms-11-00105-s001.zip › microorganisms-2094831-supplementary.pdf]

The ItOSS-Regional Working Group: Serena Donati, Alice Maraschini, Edoardo Corsi Decenti, Paola D'Aloja, Ilaria Lega (National Centre for Disease Prevention and Health Promotion, Istituto Superiore di Sanità – Italian National Institute of Health, Rome, Italy), Paolo Accorsi (Department of Obstetrics and Gynecology, AUSL Modena, Modena, Italy), Maria Paola Belluomini (Unit of Obstetrics and Gynecology, San Luca Hospital, Lucca, Italy), Marisa Biasio (Department of Obstetrics and Gynecology, University of Turin, Turin, Italy), Maria Bisulli (Obstetric Unit, University Hospital of Bologna, Bologna, Italy), Alessandra Bongiovanni (Unit of Obstetrics and Gynecology, Maggiore SS Savigliano Hospital, Cuneo, Italy), Paola Del Carlo (Unit of Obstetrics and Gynecology, San Giovanni di Dio Torregalli Hospital, Florence, Italy), Maddalena Casarotto (Unit of Obstetrics and Gynecology, Pordenone Hospital, Pordenone, Italy), Maria Grazia Castiglione (Maternal Child Department, Gynaecology and Obstetrics Unit, ARNAS Garibaldi Hospital, Catania, Italy), Maria Catania (Unit of Obstetrics and Gynecology, Buccheri La Ferla Hospital, Palermo, Italy), Irene Cetin (Department of Biomedical and Clinical Sciences, University of Milan, Milan, Italy; Department of Obstetrics and Gynecology, V. Buzzi Hospital, ASST Fatebenefratelli Sacco, Milan, Italy), Elisabetta Colciago (University of Milan-Bicocca School of Medicine and Surgery, Monza, Italy), Venere Coppola (Obstetric Unit, Misericordia Hospital, Grosseto, Italy), Gabriella Dardanoni (Department for Health Activities and Epidemiological Observatory Sicily Region, Palermo Italy), Paola Del Carlo (Unit of Obstetrics and Gynecology, San Giovanni di Dio Torregalli Hospital, Florence, Italy), Lorenza Driul (Department of Medicine, University of Udine, Gynecology and Obstetrics Clinic, Udine, Italy), Donatella Fossa (Department of Obstetrics and Gynecology, Sant'Anna Hospital-ASST Lariana, Como, Italy), Isabella Marzia Maini (Department of Obstetrics and Gynecology, Division of Obstetrics, Foundation MBBM Onlus at San Gerardo Hospital, Monza, Italy), Alessia Marras (Department of Molecular and Developmental Medicine, Division of Obstetrics and Gynecology, University of Siena, Siena, Italy), Federica Mariuzzo (Department of Obstetrics and Gynecology, Treviglio Hospital, ASST Bergamo Ovest, Bergamo, Italy), Gianpaolo Maso (Institute for Maternal and Child Health, IRCCS Burlo Garofolo, Trieste, Italy), Luisa Mondo (Department of Epidemiology, ASL TO3 Piedmont Region, Turin, Italy), Elisabetta Nelli (Department of Maternal and Child Health, A. Manzoni Hospital, Lecco, Italy), Giulia Pedrielli (Mother-Infant Department, University of Modena and Reggio Emilia, Modena, Italy), Monia Puglia (Regional Health Agency, Tuscany, Italy), Raffaella Rusciani (Department of Epidemiology, ASL TO3 Piedmont Region, Turin, Italy), Pasqualina Russo (Unit of Obstetrics

and Gynecology, Ciriè Hospital, Turin, Italy), Viola Seravalli (Department of Health Sciences, Obstetrics and Gynecology Unit, University of Florence, Florence, Italy), Daniela Spettoli (SaPeRiDoC - Documentation Centre on Perinatal and Reproductive Health, Primary Care Service, Regional Health Authority of Emilia-Romagna, Bologna, Italy), Giliana Ternelli (Obstetrics and Gynecology Unit, Department of Medical and Surgical Sciences for Mother, Child and Adult, University of Modena and Reggio Emilia, Modena, Italy), Elisabetta Venegoni (Unit of Obstetrics and Gynecology, Magenta Hospital, Milan, Italy), Fabio Voller (Regional Health Agency, Tuscany, Italy),
